# Rheological Properties of Commercially Available Hyaluronic Acid Products in the United States for the Treatment of Osteoarthritis Knee Pain

**DOI:** 10.1177/1179544117751622

**Published:** 2018-01-03

**Authors:** Mathew Nicholls, Ajay Manjoo, Peter Shaw, Faizan Niazi, Jeffrey Rosen

**Affiliations:** 1 Department of Orthopedics and Sports Medicine, Virginia Mason Medical Center, Seattle, WA, USA; 2 Division of Orthopaedic Surgery, McMaster University, Hamilton, ON, Canada; 3 Ferring Pharmaceuticals Inc., Parsippany, NJ, USA; 4 Department of Orthopaedics & Rehabilitation, NewYork Presbyterian/Queens; Departments of Orthopaedic Surgery and Rehabilitation Medicine, Weill Cornell Medical College of Cornell University, New York, NY, USA

**Keywords:** Hyaluronic acid, viscosupplementation, knee, osteoarthritis, rheological properties

## Abstract

**Objective:**

The inconsistent results within the current literature regarding the efficacy of intra-articular-hyaluronic acid (IA-HA) for the treatment of knee osteoarthritis (OA) have been suggested to be due to intrinsic differences between individual HA products. The purpose of this investigation is to define the rheological differences between currently available HA products in the United States at the time of this study for the treatment of knee OA, which will help elaborate on the appropriateness of classifying HA products as a class opposed to as individual agents.

**Methods:**

The rheological parameters for Euflexxa, Orthovisc, Supartz, Monovisc, Synvisc, Synvisc-One, Gel-One, and Hyalgan were obtained with a TA AR 2000 EX Rheometer with a cone-plate geometry (40-mm plate diameter and a 2° cone angle) at room temperature.

**Results:**

The bulk rheological parameters of the different products suggest molecular structures traversing the range of dilute solution (Hyalgan, Supartz), semidilute solution (Euflexxa, Orthovisc), entangled solutions (Monovisc, Synvisc, Synvisc-One), and even gel-like (Gel-One) behavior.

**Conclusions:**

Due to the differences in rheological properties between IA-HA products, the universal assessment of these products as a class may not be appropriate. Instead, it may be more appropriate to assess each product individually. Future research should aim to link these differences in rheological properties to the differences in clinical efficacy seen across these IA-HA products.

## Introduction

Knee osteoarthritis (OA) is a highly prevalent degenerative disease with a significant burden on the health care system.^1^ This disease results in cartilage degradation, joint effusion, swelling, and pain within the affected joint, often resulting in disability.^2^ Age, sex, body mass index, and injuries such as meniscus and anterior cruciate ligament tears have been reported as factors influencing the onset of OA.^3,4^ One characteristic of synovial fluid in cases of knee OA is the reduction in molecular weight distribution and concentration of hyaluronic acid (HA) within the joint. Hyaluronic acid is a viscoelastic molecule that has a number of beneficial effects within the synovial fluid, including lubrication and shock absorption, anti-inflammatory effects, chondroprotective properties, proteoglycan synthesis and scaffolding, and subchondral protection.^5^ Intra-articular (IA) injection of HA is a treatment option for knee OA, as replenishing the diminishing concentration and molecular weight distribution has demonstrated clinical relief for patients.^6^ There have been varying and inconsistent results with respect to the benefits of IA-HA injection, which have been suggested to be a result of the differences in intrinsic properties between specific IA-HA products.^7,8^ Given the varied response rates between the different HAs, one should seek to better differentiate between that available products in both rheological properties and biological activity.

Viscoelastic fluids exhibit zero shear rate viscosity (ability to resist permanent deformation under long-term loading), steady shear rates (testing the viscosity during constant shear of the fluid), a characteristic relaxation time (λ—the time it takes to recover to its original state following deformation) estimated as the inverse of the oscillating frequency at which the elastic modulus (G′) = loss modulus (G″), as well as a plateau and a terminal region during a frequency sweep within the linear viscoelastic range (the range in which observed viscoelastic properties are independent of the forces subjected to the fluid).^9,10^ Kinematics within the knee typically oscillate between a high load, low shear rate state (squeezing flow), and a low load, high shear rate state (hydrodynamic lubricating flow). The purpose of the IA-HA product is to maintain a fluid film between the knee cartilage surfaces throughout this oscillation that precludes cartilage contact. During squeezing flow, the HA product must demonstrate an elastic response capable of preventing cartilage contact.^9,11^ During hydrodynamic lubricating flow (from tibia and femur sliding across one another), the HA product must provide a viscosity large enough to support a thin film to separate the cartilage surfaces while still allowing the cartilage surfaces to move across one another at low drag forces.^11^

Polymer solutions such as HA in the synovial fluid (SF) can act as 3 distinct solution regions: dilute, semidilute, and entangled.^12^ Healthy human synovial fluid is typically classified in the semidilute region.^13^ The polymer solution regions are characterized by molecular structures that are dependent on molecular weight distribution, concentration, interaction between polymer chains, and the solvent-polymer interactions. The corresponding region of SF is dependent on both concentration and molecular weight distribution, as a greater concentration and/or molecular weight will create a shift toward entanglement.^12^ In the dilute region, the polymer chains are in a random coil state and do not touch each other. In the semidilute region, the polymer chains begin to interact with each other, whereas entangled solutions demonstrate intermolecule interactions that affect the large-scale motions of the polymer chains.^14^

Oscillatory frequency provides additional information about the structure-property relationship of a polymer solution, as it used to determine the viscous and elastic properties of the sample and associated solution classification.^15^ In the dilute region, the behavior of the solution is primarily defined by the viscous response at a wide range of frequencies. As the solution concentration and molecular weight distribution increase, the high-frequency response is defined by the elastic modulus, and the crossover frequency shifts toward lower frequencies or larger relaxation times. Furthermore, at the entanglement region, the elastic modulus reaches a plateau at which point it is independent of frequency and behaves more like an elastic solid.^15^

The inconsistent results within the current literature regarding the efficacy of IA-HA for the treatment of knee OA have been suggested to be due to intrinsic differences between individual HA products. Some IA-HA products are linear chain (Supartz, Hyalgan, Euflexxa, and Orthovisc) and some are mixtures of linear chain and chemically cross-linked HA (Synvisc, Synvisc-One, and Gel-One). The purpose of this investigation is to define the rheological differences between currently available HA products for the treatment of knee OA, which will help elaborate on the appropriateness of classifying HA products as a class opposed to as individual agents.

## Methods

**Figure 1.**
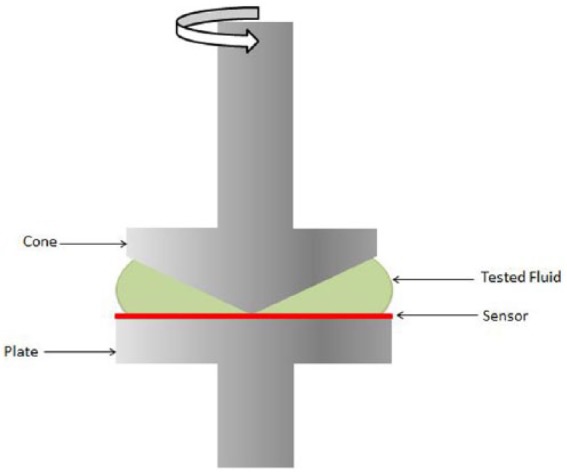
Rheometer diagram.

The rheological parameters for Euflexxa, Orthovisc, Supartz, Monovisc, Synvisc, Synvisc-One, Gel-One, and Hyalgan were obtained with a TA AR 2000 EX Rheometer with a cone-plate geometry (40-mm plate diameter and a 2° cone angle) at room temperature (Figure 1). The parameters collected for each product were concentration, molecular weight (taken from package inserts), polydispersity, elastic modulus (G′) and loss modulus (G″) as a function of oscillatory frequency (f) at both 0.5 Hz (representative of walking) and 2.5 Hz (representative of running), crossover frequency (f_c_) (defined as the frequency at which G′ = G″, also represented as relaxation time), viscosity (η) as a function of shear rate (γ), and shear rate at onset of shear thinning. Data from the measurements were independently presented and interpreted for each HA product.

The frequency sweeps were performed at strain amplitudes that were determined to be in the linear viscoelastic range. Only the viscosity data that reached equilibrium defined by the TA Rheometer algorithm were considered in the shear rate experiments. The viscosupplements were kept at room temperature prior to use. The shear rate ranges explored were 0.001 to 1000 s^−1^ and the oscillatory frequency range was 0.001 to 100 Hz, whereas the actual maximum and minimum ranges were determined by the ability of the rheometer to reach a stable reading. Each test run was duplicated with a fresh sample. Following each test, the potential for evaporation or degradation to confound the results was tested by repeating a strain sweep at 1 Hz.

## Results

### Molecular weight and concentration

Hyalgan and Supartz had the lowest molecular weights of 500 to 730 kDa^16^ and 620 to 1170 kDa,^17^ respectively. Monovisc and Orthovisc had molecular weights of 1000 to 2900 kDa^18,19^ (of the soluble portion for Monovisc), and Euflexxa was 2400 to 3600 kDa. Synvisc/Synvisc-One reported a composite molecular weight of entangled and straight chain molecules of 6000 kDa,^20,21^ whereas Gel-One did not record a molecular weight.^22^ Product concentrations ranged from 0.8 mg/mL (Synvisc and Synvisc-One) to 2.2 mg/mL (Monovisc). A complete overview of molecular weight and concentration data for the included products is provided in Table 1. All molecular weight values for IA-HA products were obtained from package inserts provided by each manufacturer and were not measured independently for this study.

**Table 1.** Steady shear rate.

	η_0.1_, Pa s	η_250_, Pa s	η_0.1_/η_250_	Mw, kDa	Concentration, mg/mL
Hyalgan	0.27	0.12	2.33	500-730	1
Supartz	3.07	0.28	10.9	620-1170	1
Monovisc	56.4	1.10	51.5	1000-2900^a^	2.2
Orthovisc	120.8	0.71	170.4	1000-2900	1.5
Euflexxa	91.2	0.39	237.2	2400-3600	1
Gel-One	190.2	0.78	243.0	NA^b^	1
Synvisc	191.7	0.26	740.7	6000^c^	0.8
Synvisc-One	184.4	0.28	651.2	6000^c^	0.8

Abbreviations: η_0_, zero shear rate viscosity; η_250_, viscosity at a 250 s^−1^; η_0.1_/η_250_, viscosity shear rate ratio; Mw, molecular weight; NA, not applicable.

a Molecular weight of monomer, not total polymer in solution.

b Not reported as formulation is cross-linked.

c Only reflective of the soluble portion.

### Steady shear rate

**Figure 2.**
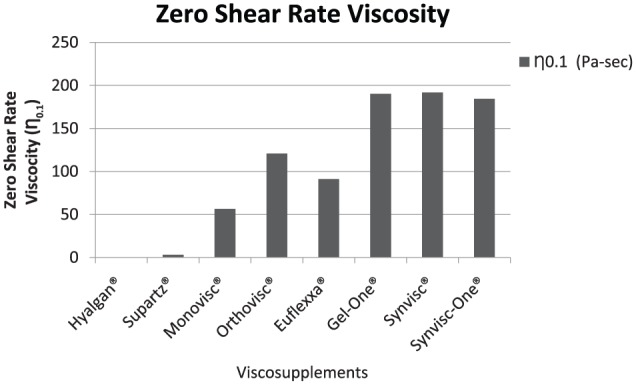
Zero shear rate viscosity (η_0.1_) of different viscosupplements.

**Figure 3.**
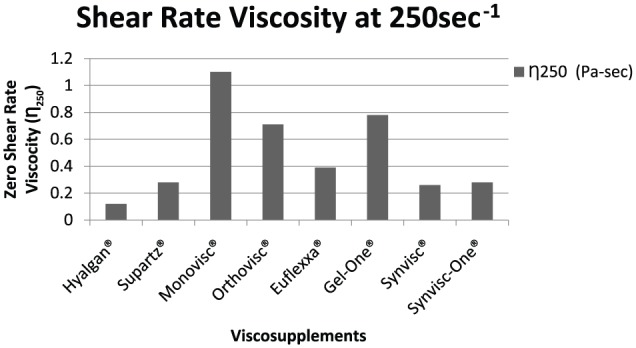
Shear rate viscosity (η_250_) of different viscosupplements.

**Figure 4.**
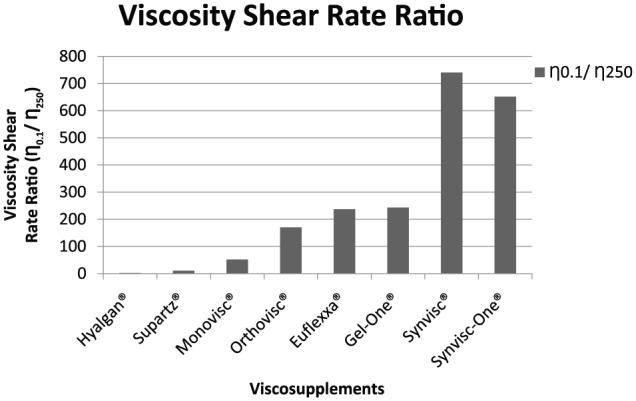
Viscosity shear rate ratio (η_0.1_/η_250_) of different viscosupplements.

The zero shear rate viscosities (η_0.1_) of the tested products ranged from 0.27 Pa s for Hyalgan to 191.7 Pa s for Synvisc, respectively (Figure 2). Viscosity at a 250 s^−1^ shear rate ranged from 0.12 Pa s for Hyalgan to 1.10 Pa s for Monovisc (Figure 3). The shear rate ratios of viscosity at 0.1 and 250 s^−1^ (η_0.1_/η_250_) ranged from 2.33 for Hyalgan to 740.7 for Synvisc (Figure 4). Steady shear rate data for all products is included within Table 1.

### Elastic and loss moduli

The tested products’ elastic modulus at 0.5 Hz (representative of walking) ranged from 0.03 for Hyalgan to 94.6 for Synvisc-One, whereas the elastic modulus at 2.5 Hz (representative of running) ranged from 11.8 for Supartz to 147.4 in Orthovisc. The 2.5-Hz elastic modulus could not be measured for Hyalgan. The products’ loss modulus at 0.5 Hz ranged from 0.69 for Hyalgan to 57.2 for Orthovisc, whereas the products’ 2.5-Hz loss modulus ranged from 5.9 for Gel-One to 85.0 for Monovisc. The loss modulus for Hyalgan could not be measured at 2.5 Hz. A complete overview of product elastic and loss moduli at 0.5 and 2.5 Hz is included in Table 2. All tested products except for Gel-One demonstrated a terminal region in which the loss modulus exceeded the elastic modulus.

**Table 2.** Oscillatory sweep.

	f_c_, Hz	λ_r_, s	G′ at 0.5 Hz	G″ at 0.5 Hz	G′ at 2.5 Hz	G″ at 2.5 Hz
Hyalgan	>10^a^	<0.01^a^	0.03	0.69	—	—
Supartz	3.98	0.25	2.2	6.5	11.8	18.1
Monovisc	2.51	0.4	34.2	41.5	91.2	85.0
Orthovisc	0.16	6.26	79.5	57.2	147.4	68.2
Euflexxa	0.10	10	57.4	32.1	93.2	34.8
Gel-One	—	—	11.0	3.2	15.0	5.9
Synvisc	<0.01^a^	>100^a^	79.9	20.3	99.2	17.8
Synvisc-One	<0.01^a^	>100^a^	94.6	22.3	115.1	18.8

Abbreviations: λ_r_, relaxation time; f_c_, crossover frequency; G′, elastic modulus; G″, loss modulus.

a Extrapolated.

— indicates could not be measured.

### Crossover frequency and relaxation times

The crossover frequencies of the tested products ranged from Hyalgan, which was extrapolated to have a crossover frequency of >10 Hz, to Synvisc and Synvisc-One, which had extrapolated crossover frequencies of <0.01 Hz. A crossover frequency could not be determined for Gel-One. The corresponding characteristic relaxation times of the products ranged from <0.01 second for Hyalgan to >100 seconds for Synvisc and Synvisc-One. A relaxation time could not be determined for Gel-One. A complete summary of product crossover frequencies and relaxation times is provided within Table 2.

## Discussion

The results of this analysis demonstrated the differences between available IA-HA products with respect to rheological properties. Tested products varied greatly with respect to their polymer solution characteristics, which ranged from dilute solutions to elastic-solid characteristics. Hyalgan demonstrated the rheological properties of a dilute solution, as it had a weak shear thinning response to shear rate and a relaxation time much faster than the other products. Supartz was shown to be a product that was approaching the semidilute region. The shear thinning response was more robust, and the crossover frequency was shifted to lower frequencies (3.98 Hz); however, the solution did not demonstrate a plateau for G′ (as seen in Table 2: G′ at 2.5 Hz = 11.8). Orthovisc and Euflexxa both exhibited characteristics of a semidilute solution, as there was a robust shear thinning response at high shear rates, relaxation times on the order of seconds, a flattening of the G′ beyond the crossover frequency (as seen in crossover frequencies and G′ within Table 2), and a wider range of linear viscoelastic response dominated by the elastic component. Monovisc did not exhibit a Newtonian plateau in viscosity and demonstrates purely power law behavior and a relatively high G′, indicative of the presence of entanglements within this highly HA concentrated solution (as seen in Table 2, G′ at 0.5 Hz = 34.2 and a corresponding crossover frequency of 2.51). Synvisc and Synvisc-One also did not exhibit a Newtonian viscosity plateau (very low crossover frequency and high G′ values) and are observed to be highly shear thinning as seen from the η_0.1_/η_250_ ratio (Table 1). Both formulations show an extrapolated crossover frequency at less than 0.01 Hz, representing a relaxation time greater than 100 seconds. Synvisc and Synvisc-One also have the highest molecular weight and are known to be partially cross-linked. The oscillatory sweep response shows a distinct plateau in G′ in comparison with the formulations discussed above exhibiting the characteristics of an entangled solution in which G′ is independent of frequency. Gel-One rheology is typical of a cross-linked gel. It exhibits only shear thinning behavior in steady shear and does not exhibit a terminal region where G″ > G′ but exhibits a flat region where G′ > G″ and both are independent of frequency. Gel-One behaves as an elastic solid in the linear viscoelastic deformation region at the frequencies studied.^15^

This study was strengthened by its robust assessment of rheological properties for individual IA-HA products using commonly used and accepted methods of rheological property testing.^15^ This study is limited by the duplicate testing method conducted, as triplicate testing is typically considered a more robust method of testing. The use of 2 samples from the same manufacturing lot is an additional limitation. This study is limited by the lack of clinical implications with respect to the treatment of knee OA, as these results do not provide insight into the efficacy of the tested products. Despite this limitation, these results provide valuable insight into the differences in basic properties of available IA-HA products. This adds validity to the assertion that not all IA-HA products are similar and thus should not be assessed as a class but as individual agents instead. The shear thinning response differences between high and low molecular weights, as well as cross-linked and non–cross-linked products, require further investigation with respect to clinical implications under physiological friction forces within the knee joint.

## Conclusions

The rheological characterization of the tested IA-HA products showed that the solutions differ in their response to linear and nonlinear deformations. The bulk rheological parameters of the different products suggest molecular structures traversing the range of dilute solution (Hyalgan, Supartz), semidilute solution (Euflexxa, Orthovisc), entangled solutions (Monovisc Synvisc, Synvisc-One, and even gel-like (Gel-One) behavior. Due to the differences in rheological properties between IA-HA products, the universal assessment of these products as a class may not be appropriate. Instead, it may be more appropriate to assess each product individually. Future research should aim to link these differences in rheological properties to the differences in clinical efficacy seen across these IA-HA products.
